# Microsaccades enable efficient synchrony-based visual feature learning and detection

**DOI:** 10.1186/1471-2202-15-S1-P121

**Published:** 2014-07-21

**Authors:** Timothée Masquelier, Geoffrey Portelli, Pierre Kornprobst

**Affiliations:** 1 Institut de la Vision, UPMC Université Paris 06, Paris, 75012, France; 2 CNRS, UMR 7210, Paris, 75012, France; 3 Neuromathcomp Project Team, Inria Sophia Antipolis Méditerranée, 06902, France

## 

Fixational eye movements are common across vertebrates, yet their functional roles, if any, are debated [1]. To investigate this issue, we exposed the Virtual Retina simulator [2] to natural images, generated realistic drifts and microsaccades using the model of ref. [3], and analyzed the output spike trains of the parvocellular retinal ganglion cells (RGC).

We first computed cross-correlograms between pairs of RGC that are strongly excited by the image corresponding to the mean eye position. Not surprisingly, in the absence of eye movements, that is when analyzing the tonic (sustained) response to a static image, these cross-correlograms are flat. Adding some slow drift (~20 min/s, self-avoiding random walk) creates long timescale (>1s) correlations because both cells tend to have high firing rates for central positions. Adding microsaccades (~0.5° in 25ms, that is ~20°/s) creates short timescale (tens of ms) correlations: cells that are strongly excited at a particular landing location tend to spike synchronously shortly after the landing.

What do the patterns of synchronous spikes represent? To investigate this issue, we fed the RGC spike trains to neurons equipped with spike timing-dependent plasticity (STDP) and lateral inhibitory connections, as in ref. [4]. Neurons self-organized, and each one selected a set of afferents that consistently fired synchronously. We then reconstructed the corresponding visual stimuli by convolving the synaptic weight matrices with the RGC receptive fields. In most cases, we could easily recognize what was learned (e.g. a face), and the neuron was selective (e.g. only responded for microsaccades that landed on a face). Without eye movements, or with only the drift, the STDP-based learning failed, because it needs correlations at a timescale roughly matching the STDP time constants [5].

Microsaccades are thus necessary to generate a synchrony-based coding scheme. More specifically, after each microsaccade landing, cells that are strongly excited by the image corresponding to the landing location tend to fire their first spikes synchronously. Patterns of synchronous spikes can be decoded rapidly – as soon as the first spikes are received – by downstream “coincidence detector” neurons, which do not need to know the landing times. Finally, the required connectivity to do so can spontaneously emerge with STDP. As a whole, these results suggest a new role for microsaccades – to enable efficient visual feature learning and detection thanks to synchronization – that differs from other proposals such as time-to-first spike coding with respect to microsaccade landing times.
